# Analyzing Complexity and Fractality of Glucose Dynamics in a Pregnant Woman with Type 2 Diabetes under Treatment

**DOI:** 10.7150/ijbs.33825

**Published:** 2019-09-07

**Authors:** Xiaoyan Chen, Dandan Wang, Jinxiang Lin, Teng Zhang, Shunyou Deng, Lianyi Huang, Yu Jin, Chang Chen, Zhaozhi Zhang, Jun Zheng, Baoqing Sun, Paul Bogdan, Xiaohua Douglas Zhang

**Affiliations:** 1 Department of Endocrinology, First Affiliated Hospital of Guangzhou Medical University, Guangzhou 510000, China; 2 Faculty of Health Sciences, University of Macau, Taipa 999078, Macau; 3 Department of Statistical Science, Duke University, Durham, NC 27708, USA; 4 Department of Allergy and Clinical Immunology, Guangzhou Institute of Respiratory Diseases, State Key Laboratory of Respiratory Disease, National Clinical Research Center for Respiratory Disease, First Affiliated Hospital of Guangzhou Medical University, Guangzhou 510000, China; 5 Department of Electrical Engineering - Systems, University of Southern California, CA 90089, USA

**Keywords:** Continuous glucose monitoring, Complexity analysis, Multiscale sample entropy, Fractal analysis, Type 2 diabetes with pregnancy

## Abstract

Currently, the rapid development of continuous glucose monitoring (CGM) device brings new insights into the treatment of diabetic patients including those during pregnancy. Complexity and fractality have recently under fast development for extracting information embodied in glucose dynamics measured using CGM. Although scientists have investigated the difference of complexity in glucose dynamics between diabetes and non-diabetes in order to discover better approaches for diabetes care, no one has analyzed the complexity and fractality of glucose dynamics during the process of adopting CGM to successfully treat pregnant women with type 2 diabetes. Thus, we analyzed the complexity and fractality using power spectral density (PSD), multi-scale sample entropy (MSE) and multifractal detrended fluctuation analysis (MF-DFA) in a clinical case. Our results show that (i) there exists multifractal behavior in blood glucose dynamics; (ii) the alpha stable distribution fits to the glucose increment data better than the Gaussian distribution; and (iii) the “global” complexity indicated by multiscale entropy, spectrum exponent and Hurst exponent increase and the “local” complexity indicated by multifractal spectrum decrease after the successful therapy. Our results offer findings that may bring value to health care providers for managing glucose levels of pregnant women with type 2 diabetes as well as provide scientists a reference on applying complexity and fractality in the clinical practice of treating diabetes.

## Introduction

Diabetes mellitus is a chronic metabolic disease associated with long-term damage to various organ systems 1. It causes many complications including cardiovascular diseases, nephropathy, stroke and retinopathy 1, 2. Moreover, diabetes with pregnancy is more complicated since hyperglycemia can lead to congenital malformations, preterm delivery, preeclampsia, macrosomia, shoulder dystocia, cesarean delivery and maternal mortality 3. Currently, the rapid development of continuous glucose monitoring (CGM) device brings new insights into the treatment of diabetic patients including those during pregnancy.

Traditionally, in clinical practice, the information extracted from CGM devices is primarily based on calculations of the percentage of time above and below given thresholds, range, and average values of blood glucose, but largely ignores the dynamics of glucose fluctuations 4, 5. To extract the information encoded in the dynamical structure of glucose fluctuations, recent research developments in this area employ fractal analysis such as detrended analysis and MSE to assess the complexity of CGM data. Scientists have investigated the complexity of glucose dynamics in type 2 diabetes 4, type 1 diabetes 6, and a mixed pool of type 1 and 2 diabetes 7 against non-diabetics and found that non-diabetic people have a higher complexity than patients with diabetes. However, no one has analyzed the complexity 4, 8-10 and fractality 5, 11-16 of glucose dynamics during a therapeutic treatment. In this paper, we analyzed the complexity and fractality of glucose dynamics in a pregnant woman with type 2 diabetes during her therapeutic treatment.

### Materials and methods

Nonlinear metrics have been a key category of quantity for extracting information embodied in the dynamics of physiological signals 8-10. Here we adopted the following three non-linear statistical parameters to analyze the complexity and fractality of glucose dynamics.

### Power spectral density

Fourier transform can convert a time series from time domain to frequency domain and obtain the frequency distribution of the time series. The power spectrum function |***A***(***f***)|**^2^** represents the power of harmonics with frequency ***f***. The PSD of time series describes the distribution of signal power in the frequency domain. For the fractal time series, the relationship between power and frequency is:


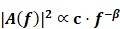
 (1)

where c is a constant. c represents the amplitude of the harmonics with frequency ***f*** and the ***β*** is the spectral exponent (the negative slope of the line on the plot of log power vs. log frequency). For fractal Gaussian noises which are stationary time series, -**1**<***β***<**1** while for fractal Brownian motions which is non-stationary time series, <***β***<**3**.

The power-law relationship of power and frequency has been found in many physiological time series, such as blood cell perfusion time series 17, heart rate variability 18 and blood glucose. The spectral exponent is the most common parameter for fractal analysis of time series 19. The method we use to calculate the ***β*** is lowPSD*_we_* which gives the best performance for spectrum analysis 17. The high-frequency region of spectrum (fs/8<f<fs/2) is excluded before the linear fitting of log-log plot.

The power-law relationship of power and frequency has been found in many physiological time series and the spectral exponent has been found to change during different physiological or pathological conditions such as disease and aging 17, 20, 21.

### Multiscale entropy

Sample entropy is a measure of complexity or irregularity, which was first proposed by Richman and Moorman 22. The algorithm for calculating sample entropy is as follows. Let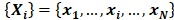 represent a time series of length N. Define the template vector of length *m*:


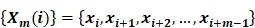
 (2)

and the distance function:


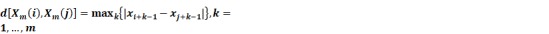
 (3)

Count the number of vector pairs in template vectors of length *m* and *m+1* having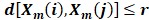 and denote it by *B* and *A*, respectively. Then the sample entropy is defined as:


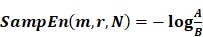
 (4)

where *A* is the number of template vector pairs having 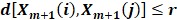 of length *m+1*, and *B* is the number of template vector pairs having of length m. Thus, 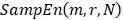 is the negative natural logarithm of the conditional probability that two sequences similar for *m* points remain similar at the next point with a tolerance *r*.

With the concept of sample entropy, the process for MSE analysis is as follows. First divide the original signal represented by a time series 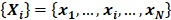 into nonoverlapping segments of equal length (*k*) and calculate the mean value of the data points in each of these segments. This process is called coarse graining, and the newly generated time series is called coarse-grained time series. The length *k* is called a scale factor. The coarse-graining process is repeated for multiple values of *k*. As the scale factor *k* changes, we construct different coarse-grained time series, and subsequently we calculate corresponding entropy values on the newly coarse-grained time series. The entropy of coarse-grained time series can be plotted against the scale factor *k*. An R package for calculating MSE has recently been developed 10. Here that R package was used to calculate MSE in our clinical case.

### Multifractal detrended fluctuation analysis

The complexity of time series can also be expressed as multifractal behaviors. MF-DFA is a method proposed by Kantelhardt et al. to detect the multifractal behaviors in non-stationary time series. MF-DFA explores the scaling behaviors of time series for different values of *q* which is the order of fluctuation and determines the q-order generalized Hurst exponent *H*(*q*). Generally, the smaller *H*(*q*) describes the scaling behaviors of large fluctuations, while the larger *H*(*q*) describes the scaling behaviors of small fluctuations 23. When *q*=2, MF-DFA corresponds to the ordinary detrended fluctuation analysis 24. The *H*(2) is the scaling exponent of detrended fluctuation analysis and the classical Hurst exponent relates to *H*(2). The multifractal spectrum *D*(*q*) is another measurement of multifractal behaviors and can be obtained via the Legendre transform. The multifractal spectrum will be a single-humped shape with a large arc for multifractal time series. The width of multifractal spectrum Δ*h* is calculated by:


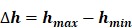
 (5)

which can reflect the degree of multifractal behaviors.

The MF-DFA has been proved to be more reliable than other methods of multifractal analysis for time series 25 and there have been a few attempts at applying it to higher dimension 26. Mukli proposed a new method for multifractal analysis based on MF-DFA 27. The development of MF-DFA has prompted new applications to complexity of physiological time series 28-31. Here we use the Matlab code created by Ihlen to analyze the glucose data.

### Clinical case

We observed a 34-year-old woman in early pregnancy who had suffered from both type 2 diabetes and hypertension for 3 years. Her physical examination revealed very high glucose levels (fasting pasma glucose (FPG) 14.3mmol/L and glycosylated hemoglobin A1c (HBA1c) 9.6%) with positive urine ketone and urine protein, but normal levels in estrogen, progesterone and human chorionic gonadotropin. The patient signed the informed consent form.

The patient first received the treatment of multiple dose injection for 7 days without efficacy of glucose control (FPG 10-12.9mmol/L, 2-hour postprandial blood glucose (2hPG) 11-14.4mmol/L, positive urine ketone). Subsequently, she was treated with a therapy of continuous subcutaneous insulin infusion based on CGM for the first time from 11:55, June 20, 2017 to 08:45, June 23, 2017. This treatment period is referred to as Period 1 throughout the study. The glucose reading from the sensors did not start at 0:00 but at around 12:00. The insulin dose was adjusted after lunch every day based on the glucose readings in the past 24 hours. The basal rates and meal bolus are displayed in Table S1. The method was applied again from June 26 to June 28 because the levels of fasting glucose (7.9mmol/L to 8.4mmol/L) were still high. This treatment period is referred to as Period 2 throughout the study. After the two periods of treatment, the patient was in good condition, namely normal level of glucose (FPG 5-6.3mmol/L, 2hPG 6.5-8.5mmol/L, negative urine ketone) and normal blood pressure (120-130mmHg/70-80mmHg). Moreover, the embryo in the uterus developed well. During the integrative therapy, the diet was not altered.

The CGM based therapy in our case was applied with a device called the MiniMed Paradigm722 real time insulin pump system. This system included an insulin pump, a CGM sensor, a transmitter and a carelink personal software. One new sensor can monitor glucose continuously every five minutes for 3 days. Eight self-monitored blood glucose measurements (three pre-meal, three post-meal, one bedtime and one 3:00) were tested every day. The glucose targets were set for pre-meal, 1h post meal and 2h post meal. The targeted upper limits 32 were 5.3mmol/L, 7.8mmol/L and 6.7mmol/L respectively while the lower limit was 3.3 mmol/L for all glucose targets.

The initial total daily dose was 80% of the previous dose in the treatment of multiple dose injection. The basal dose and meal bolus were each accounting for approximately 50% of total daily dose. The three meal boluses were divided using a ratio of 1:1:1 for each meal and results were rounded up to 8U. Six basal rate segments were programmed across a 24hr period. The average basal rate was approximately one unit per hour. 10%-20% adjustment of basal rates and meal bolus was made according to the previous 24hr sensor glucose and blood glucose. The details of the application of the treatment can be referred to Figure S1 in the Supplementary Materials.

In the MSE calculation, the entropy value is depended on the embedding dimension m and the tolerance* r*. The selection of *m* and *r* can be optimized 33. Costa et al. have discussed the relationship between MSE and the parameters *m* and *r*. They obtained the most accurate results when *m=2* and *r=0.15* 34. Therefore, in this calculation, the embedding dimension m was set as 2 and the tolerance r as 0.15. When analyzing MF-DFA, in order to satisfy a linear relationship: *log*_2_*F_q_~scale*, we need to choose suitable polynomial m and scale to get the best linear fit results. Therefore, the order of the fitted polynomial *m* is set to 2 and the scale is set to 2^4^~2^9^.

## Results

### Distribution of glucose increments

**Figure 1:**
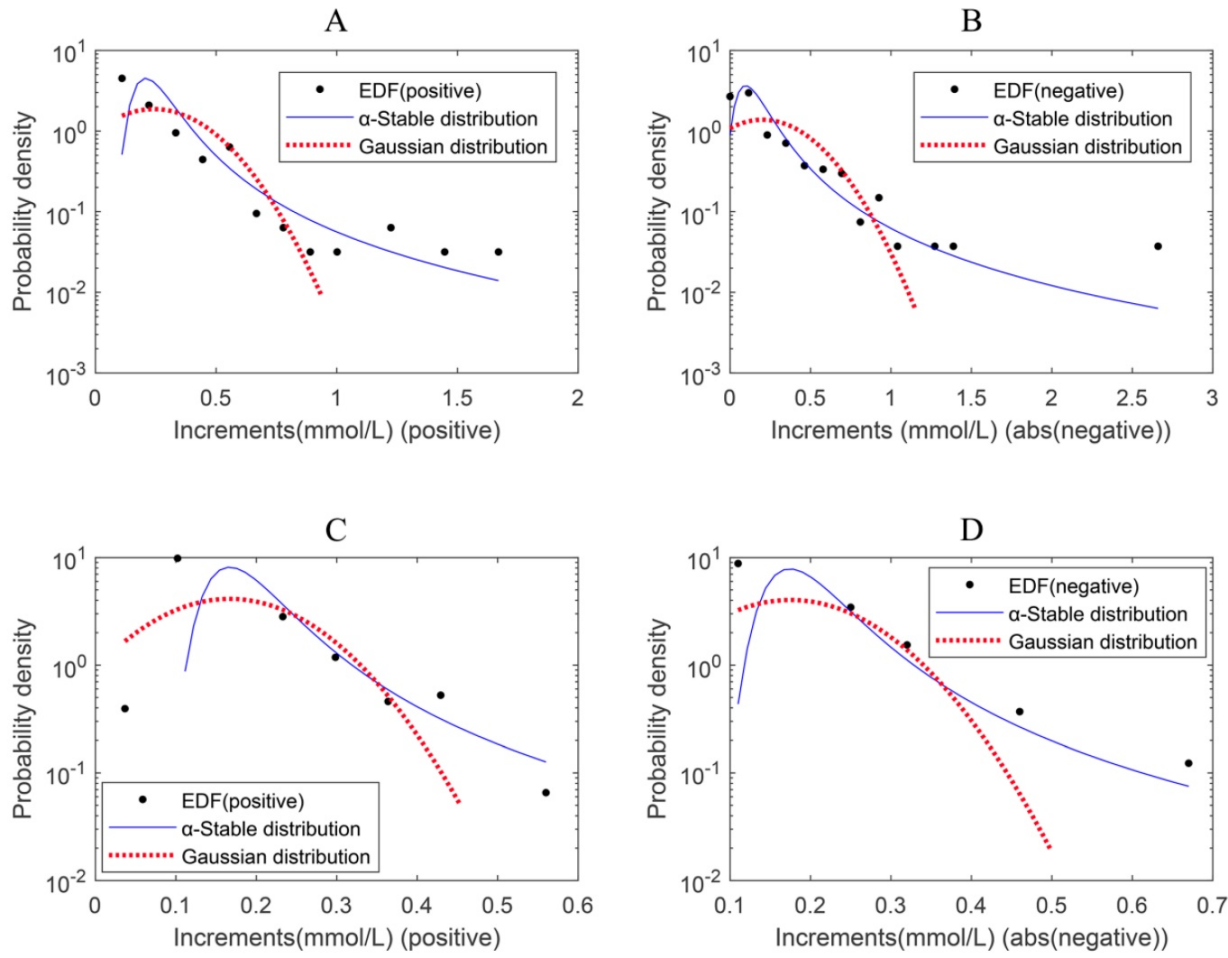
** Displaying the results of distribution fitting analysis.** (A) Distribution fitting for positive increment of blood glucose values in Period 1, α-stable (1.1430, 1, 0.061, 0.4945) and Gaussian (0.2428, 0.2126). (B) Distribution fitting for absolute values of negative increment of blood glucose values in Period 1, α-stable (1.0852, 1, 0.0759, 0.6886) and Gaussian (0.2020, 0.2887). (C) Distribution fitting for positive increment of blood glucose values in Period 2, α-stable (1.1565, 1, 0.0338, 0.3126) and Gaussian (0.1668, 0.0966). (D) Distribution fitting for absolute values of negative increment of blood glucose values in Period 2, α-stable (1.1735, 1, 0.0352, 0.3112) and Gaussian (0.1752, 0.0989). EDF: empirical density function.

**Figure 2:**
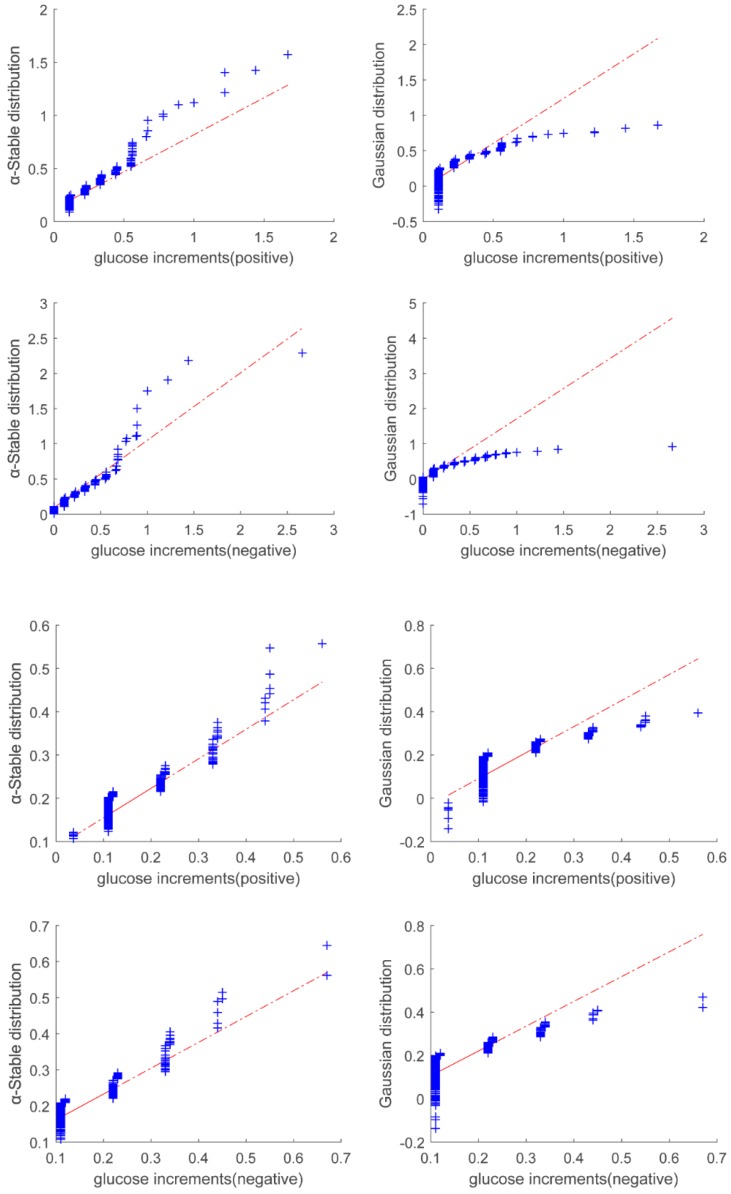
** Quantile-quantile plot of glucose increments for Period 1 (Top two panels) and Period 2 (bottom two panels).** The figure shows that the alpha stable distribution (shown in the left panels) fits to the data better than Gaussian distribution (shown in the right panels).

The empirical probability density function of the magnitude (absolute value) of the positive blood glucose increments deviates from the Gaussian distribution and is better fitted by an α-stable distribution for either Period 1 or Period 2 (Figure 1A&C and the left panels of Figure 2). This is also true for the magnitude (absolute value) of the negative blood glucose increments (Figure 1B&D and the right panels of Figure 2). Therefore, modeling blood sugar dynamics via linear state space models is inadequate and there is a need to analyze the data using non-linear methods such as complexity analysis. Consequently, we applied PSD, MSE and MF-DFA to analyze glucose dynamics in the two treatment periods.

### Change of complexity in the clinical case

**Figure 3:**
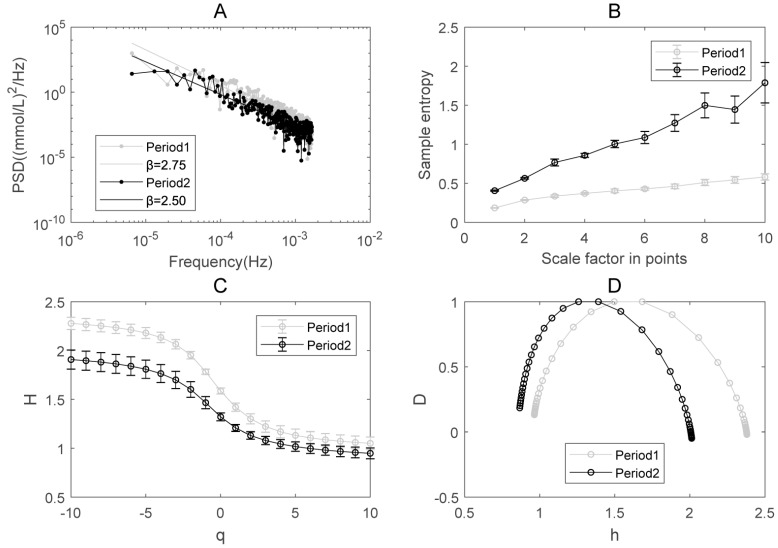
** The complexity of glucose dynamics in Periods 1 and 2 for the integrative treatment.** (A) The power spectral density. (B) Multiscale entropy analysis (MSE). (C) Multifractal detrended fluctuation analysis: Q-order Hurst exponent. (D) Multifractal spectrum analysis. The error bar (i.e., standard deviation) in Panels B and C was given by bootstrapping all the ordered glucose time series that contain 95% of the original data.

The results of PSD analysis on glucose dynamics indicate the existence of power-law relationship between the PSD and frequency in both Periods 1 and 2 (Figure 3A). The spectrum exponent β is the negative slope of the linear regression line, β=2 for Period 1 and β=1.93 for Period 2, respectively. β>1 means that the glucose readings are better approximated by a non-stationary fractional Brownian motion 16. β in Period 1 is greater than in Period 2, indicating that the decay rate of the PSD becomes slower after the integrative therapy. According to the relationship between β and *H_fBm_* (Hurst exponent), *β*=2*H_fBm_*+1 35, we can calculate *H_fBm_* in both periods. The result shows that *H_fBm_* in Period 1 (0.87+/-0.019) is also larger than that in Period 2 (0.775+/-0.036).

MSE has been applied to explore the complexity of numerous physiological signals 8, 10, 36-38. Sample entropy is a measure of irregularity and multi-scale sample entropy is a measure of complexity. The sample entropy at each scale in Period 2 is substantially higher than that in Period 1. This indicates that the complexity of glucose dynamics increased in Period 2 compared to Period 1 in the patient (Figure 3B).

The results of MF-DFA applied to the glucose dynamics indicates that the generalized Hurst exponent in Period 1 is greater than that in Period 2 (Figure 3C). The higher the Hurst exponent, the lower the fractal dimension, and the lower the global complexity accordingly in certain circumstances 39. The overall decrease of H(q), which is consistent with the results of PSD, indicates that the global complexity increased after the integrative therapy.

The single-humped shape of the multifractal spectrum further illustrates the existence of multifractal behavior in blood glucose dynamics (Figure 3D). The width of the multifractal spectrum ∆h is a measure of multifractality. Different from the sample entropy and Hurst exponent, ∆h reflects the “local” fractality and complexity of time series 27. The width of the multifractal spectrum ∆h in Period 1 (i.e., 1.32) is greater than in Period 2 (i.e., 1.12), indicating that the degree of multifractality and the “local” complexity become smaller after treatment.

## Discussion

Based on the data of glucose dynamics measured by CGM, we analyzed complexity and fractality of a pregnant woman with type 2 diabetes that was treated successfully with continuous subcutaneous insulin infusion and CGM, and compared the results in two treatment periods. After this integrative treatment, the glucose level was reduced to a normal range and became stable. The complexity analysis shows that (i) the Spectrum exponent β (the negative slope of the linear regression line in Figure 3A) decreased; (ii) the multiscale entropy increased (Figure 3B); (iii) the Hurst exponent decreased (figure 3C) and (iv) the width of the multifractal spectrum decreased (Figure 3D) in Period 2 as compared to Period 1. These results indicated that the “global” complexity indicated by multiscale entropy, spectrum exponent and Hurst exponent increased; and the “local” complexity indicated by multifractal spectrum decreased after a successful treatment on diabetes.

To the best of our knowledge, this is the first time that an increased “global” complexity in glucose dynamics during the treatment periods for diabetes was displayed in a clinical case where a pregnant woman with type 2 diabetes was treated using continuous subcutaneous insulin infusion along with CGM. Taking into consideration other studies having shown that the complexity of blood glucose systems is higher in those without diabetes compared to patients with diabetes 4, 6, 7, this gives us increased confidence that complexity (global or local) has the potential of serving as an effective index to assess efficacy in the treatment of diabetes, as well as potential measures for assessing the therapeutic efficacy of various control algorithms for artificial pancreas devices. That is, the Spectrum exponent β in PSD analysis, the sample entropy in MSE analysis, Hurst exponent H(q) and the width of the multifractal spectrum ∆h in MF-DFA all have the potential of being used as the indicator of treatment adjustment in diabetes.

Another important observation is that medical cyber-physical systems should not only aim to control for ensuring specific reference values, but also to preserve a higher degree of global complexity of physiological processes which intrinsically encapsulates the capabilities of sustaining large perturbations and adapting to environmental influences 5. The observed changes of complexity in the pregnant woman with type 2 diabetes may serve as inspiration to medical cyber physical systems design and in particular to artificial pancreas design 5. More precisely, the control algorithms in these artificial pancreas devices may consider not only the blood glucose reference values, but also measures of complexity. It has been verified that the nonlinear optimal controller based on fractal calculus concepts is superior to nonfractal controllers in the artificial pancreas design 5. The nonlinear analysis based on complexity may help artificial pancreas devices to control the blood glucose more tightly and prevent medical complications.

In this paper, we used MSE, PSD and MF-DFA to measure the complexity of glucose data. The three methods are measurement of complexity from different aspects. MSE is a method to measure complexity through irregularity across different scales. Higher MSE value means higher complexity. By contrast, PSD and MFDFA are the methods to measure complexity through fractal behavior (A higher Hurst exponent corresponds to a lower fractal dimension, and higher Hurst exponent means lower complexity accordingly 39). In addition, the scale for PSD analysis is frequency and we exclude the high-frequency region of spectrum (fs/8<f<fs/2), where fs is sampling frequency, while the scale for MFDFA spans the time axis and the scale ranges are (2^4 ~ 2^9). It means that PSD is a method for fractal analysis in frequency domain and MFDFA is a method for multifractal analysis in time domain. There may be a need to explore whether and when one method is better than others for future research.

Still there are undoubtedly certain limitations to our study. To be able to obtain reliable MSE values and fractal analysis, a large number of observed data points (usually at least a thousand) are required. Furthermore, it has recently been shown 40 that sample entropy is equivalent to conditional entropy quantified by Heaviside kernel function and depends on the properties of correlation and the type of artifacts in the signal. Future research should be conducted to explore the auto-correlation properties of glucose dynamics data and to explore the type of artifacts in the data and its impact on non-linear parameters like sample entropy.

## Supplementary Material

Supplementary methods, figures, and table.

## Abbreviations

CGM: continuous glucose monitoring
MSE: multi-scale sample entropy
PSD: power spectral density
MF-DFA: multifractal detrended fluctuation analysis
FPG: fasting plasma glucose
2hPG: 2 hours postprandial blood glucose
HbA1c: glycosylated hemoglobin A1c
