# Supplementary material for: Analyzing Complexity and Fractality of Glucose Dynamics in a Pregnant Woman with Type 2 Diabetes under Treatment
Source: Int J Biol Sci. 2019 Sep 7;15(11):2373–80. doi: 10.7150/ijbs.33825 (PMC6775315; doi:10.7150/ijbs.33825)
Supplement: Supplementary file 1 — Supplementary methods, figures, and table. [file ijbsv15p2373s1.pdf]

Analyzing Complexity and Fractality of Glucose Dynamics in a Pregnant Woman with Type 2 Diabetes under Treatment

Xiaoyan Chen[1#], Dandan Wang[2#], Jinxiang Lin[1], Teng Zhang[2], Shunyou Deng[1], Lianyi Huang[1], Yu Jin[2], Chang Chen[2], Zhaozhi Zhang[3], Baoqing Sun[4], Paul Bogdan[5] and Xiaohua Douglas Zhang[2*]

*Correspondence: Xiaohua Douglas Zhang: douglaszhang@umac.mo

Application of CGM based therapy

The overall procedure is a combination of programmed and manual adjustments. The initial determination of TDD and the basal rate and meal bolus components are programmed. The subsequent adjustment of basal rates and meal boluses are adjusted based on predetermined guidelines but also doctor experience. For example, looking at **Figure 1B**, the meal boluses during lunch on June 27 during Period 2 was reduced to 3.0. This was due to a situation where the patient had to skip lunch so the predetermined meal bolus was adjusted.

Based on our clinical experience and the published literature [1-3], we applied the integrative treatment according to the workflow as shown in **Figure S1**.

Preparation for the treatment

The patient's blood glucose levels were continuously monitored using the MiniMed Paradigm722 real time insulin pump system. Glucose levels are especially noted at pre-meal, 2h post meal, bedtime and 3:00am. Next, glucose targets were set for pre-meal, 1h post meal and 2h post meal. The targeted upper limits were 5.6mmol/L, 7.8mmol/L and 6.7mmol/L respectively while the lower limit was 3.3 mmol/L for all glucose targets.More details about the workflow are described below.

Determining the initial Total Daily Dose (TDD), basal rate and meal bolus

The initial TDD was determined next. There are three ways to calculate TDD. The first method is to set TDD equal to 0.5 to 1.0 times the patient's weight in kilograms. If the patient has received insulin injection, then TDD is equal to 0.8-1.0 times the previous insulin dose. Alternatively, TDD can be set as the average of the above two TDDs. Our patient was treated with multiple dose injection therapy (maximum daily dose was 58U) before using the integrative therapy so TDD was 0.8 times of 58 (46.4U) in order to prevent hypoglycaemia. The insulin dose is comprised of a basal dose and a meal bolus. Together they each account for around 50% of TDD. Six basal rates is a generally applied standard in clinical settings because it can help to prevent the dawn phenomenon [4]. Since the overall insulin dose was 46.4U, the overall basal rate and meal bolus was set to 23.2U. The basal rate for each period was calculated according to the table in **Figure S1**.

The three meal boluses were divided using a ratio of 1:1:1 for each meal and results were rounded up. The initial basal rates and meal boluses are showed in **Table S1** (see 20/6-21/6).

Adjusting basal rates and meal bolus

The aim in the first day of the integrative therapy is mainly to explore the sensitivity to insulin and to observe the effect of the initially determined insulin dose on patients. For future days, the basal rates and meal boluses are adjusted based on analysis of the patient's glucose levels as measured by CGM during the previous day. For our patient, since the CGM measurements began at noon, the first meal bolus she received for the purposes of adjusting was lunch on Jun 20.

There are two factors to determine whether the meal bolus should be changed. The first is whether blood glucose levels fall below your targeted levels. The second is the difference in blood glucose pre-meal and 2hr post meal. Under normal circumstances, glucose levels 2hr post meal should be higher than pre-meal glucose levels by 1.7-3.3mmol/L. Accordingly, if the blood glucose levels are above your targeted levels and your blood glucose increases by more than 3.3mmol/L, the meal bolus should be increased by 10%-20% rounded to the nearest whole number. On the other hand, if the glucose levels rise less than 1.7mmol/L, the meal bolus should be decreased by 10%-20%. So for illustration, the meal bolus for our patient during breakfast for the first 24 hrs of Period 1(Jun 21) was 8U. The pre-meal and 2 hr post meal blood glucose difference for that meal was 5.1mmol/L. Since this blood glucose increase was much greater than the expected 3.3mmol/L and the blood glucose levels at the time were much higher than our targeted levels, our subsequent meal bolus for breakfast on the second day of Period 1 was increased by 20% to 10u. All meal boluses were determined through this method as can be seen in **Figure S2**. There were slight exceptions as can be seen by the small spikes throughout the graph. Those correspond either with the patient needing to eat food outside of normal meal times and extra insulin was supplemented to compensate or the patient needing to skip a meal and therefore meal boluses were lowered to account for those skipped meals.

To know whether basal rates need to be adjusted, CGM is used to determine whether blood glucose continuously increased by 1.7mmol/L or more. If so, the basal rate is increased by either 10% or 20% for all time periods in the following 24 hrs. What this means in clinical practice is that the basal rate is adjusted by 0.1U/hr or 0.2U/hr for each of the six time periods. To determine whether the increase in basal rates should be 0.1U/hr or 0.2U/hr depends on the overall trends in the blood glucose levels during that day. Usually there are either marked peaks or dips in the blood glucose levels of a patient. If the blood glucose of a patient for 24 hrs has a few noticeable dips, then in the time period that encompasses the 2-3 hr period prior to the beginning of each dip, the basal rate is increased by 0.1U/hr. All other time periods are increased by 0.2U/hr. If the blood glucose of a patient for 24 has a few noticeable peaks, then in the time period that encompasses the 2-3 period prior to the peak, the basal rate is increased by 0.2U/hr. All other time periods are increased by 0.1U/hr. On the other hand, if the glucose levels decreased by more than 1.7mmol/L or below the glucose target, then the basal rate was reduced by10%-20%. For our patient, the initial basal rates for the first 24 hours are displayed in the first row of **Table S1**. That day there was a continuous increase in blood

glucose by more than 1.7mmol which signified that basal rates must be increased for the following 24 hrs. There were two marked low points in the blood glucose graph as can be seen in Figure 1A. They were around 14:00 and 21:00. So accordingly, the two periods of time that encompass the 2-3 blocks of time prior to this dip, in this case 7:00-12:00 and 17:00-22:00, had their basal rates increased by 0.1U/hr. The other four time periods in the 24 time span had their basal rates increased by 0.2U/hr.

Comparative results of glucose profiles between Period 1 and Period 2

From Figure S2, we can see that the overall values, sensor average, and the variety in Period 1 are all smaller than the corresponding values in Period 2. Figure S3 shows the box plot of glucose values in Period 1 and Period 2. This plot is comprised of each 24-hr glucose boxplot profile. The diabetic patient revealed a clear decrease in the median, and the first and third quartiles for glucose levels. In addition, the median of the blood glucose levels decreased and the variation became smaller as values fluctuated within a smaller range. From the above results, the integrative treatment appears to have been effective in maintaining normoglycemia in the patient.

Supplementary Figures

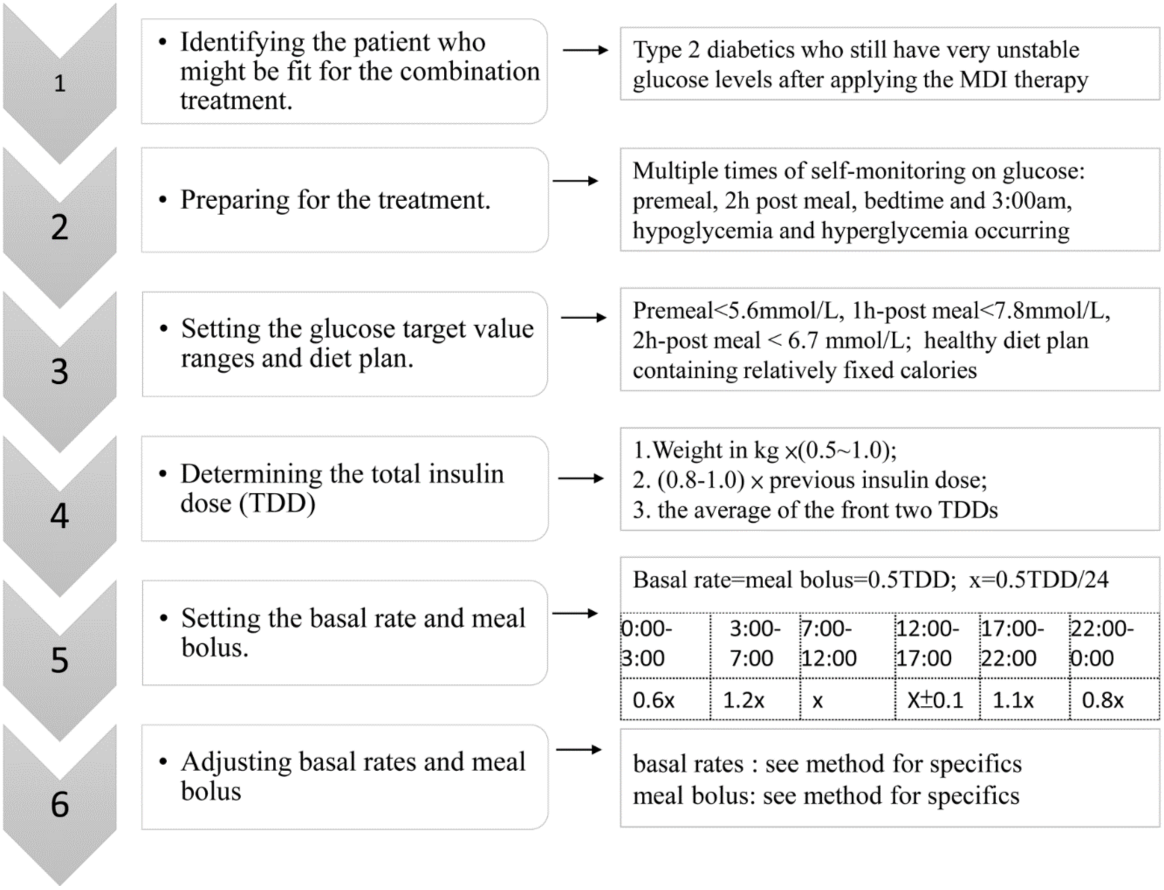

Figure S1. workflow for the application of the integrative method.

A (Period 1)

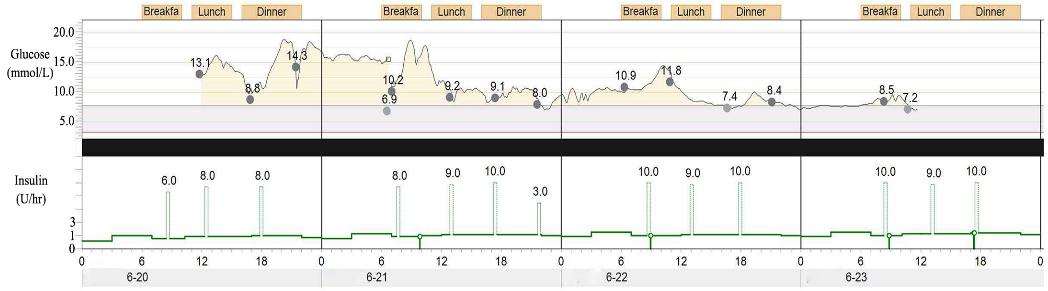

B (Period 2)

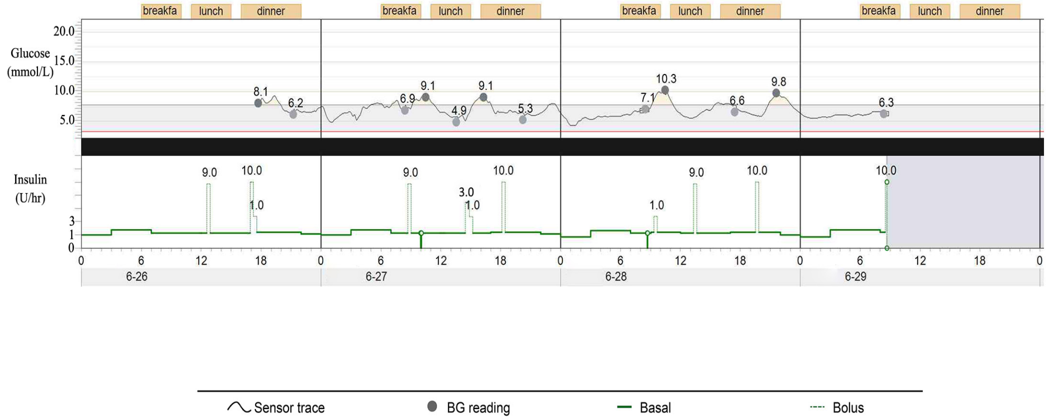

Figure S2. Trends of glucose levels and insulin dosage (basal rates and meal bolus)

(A) the graph for the first application of the integrative method of Period 1 (20/6-23/6).
(B) the graph for the second application of the integrative method of Period 2 (26/6-29/6)

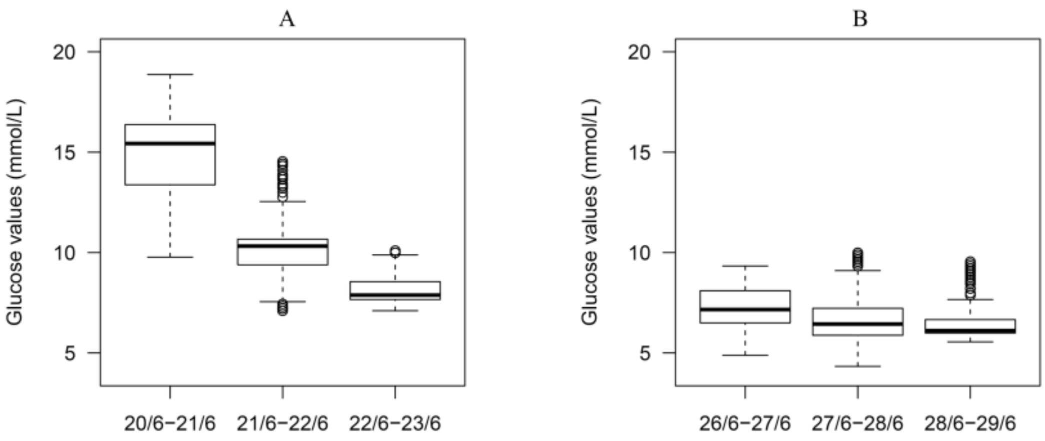

Figure S3. Box plots of glucose values for the two periods of the integrative treatment method.

(A) Box plot of glucose values for Period 1. (B) Box plot of glucose values for Period 2. A decrease in the median and the first and third quartiles for glucose levels is shown in Period 1 when compared to Period 2. In addition, the fluctuation of glucose levels in Period 2 is smaller than that in Period 1Trends of glucose levels and insulin dosage (basal rates and meal bolus).

Supplementary table

Table S1. The insulin basal rates and meal bolus programed in period 1 and period 2

Date	TDD	Basal Rate(U/hr)						Meal Bolus(U)		
	(u)	12:00-17:00	17:00-22:00	22:00-0:00	0:00-3:00	3:00-7:00	7:00-12:00	Lunch	Dinner	Breakfast
20/6-21/6	46.5	0.9	1.0	0.8	0.7	1.2	0.9	8	8	8
21/6-22/6	55.3	1.1	1.1	1.0	0.9	1.4	1.0	9	10	10
22/6-23/6	55.3	1.1	1.1	1.0	0.9	1.4	1.0	9	10	10
26/6-28/6	59.1	1.2	1.3	1.1	1.0	1.6	1.2	9	10	10

(**Note:** Since the CGM measurements began at noon, the first meal bolus she received for the purposes of adjusting was at lunch on Jun 20.**)**

Reference

1. Bruce W. Bode JK, Francine R. Kaufman. Pumping protocol: a guide to insulin pump therapy initaiation. Northridge, CA: Meditronic, Inc. 2008.
2. Medtronic. Insulin Pumps User Guide. 2008, https://wwwmedtronicdiabetescom/download-library/minimed-522-722.
3. Association EaMBoCM. China insulin pump clinical guideline. Diabetes Word. 2014; 8: 404-9.
4. Porcellati F, Lucidi P, Bolli GB, Fanelli CG. Thirty years of research on the dawn phenomenon: lessons to optimize blood glucose control in diabetes. Diabetes care. 2013; 36: 3860-2.
